# Fluid dynamics and leukocyte transit in the lymphatic system

**DOI:** 10.1093/pnasnexus/pgae195

**Published:** 2024-05-17

**Authors:** Huabing Li, Jingjing Zhang, Timothy P Padera, James W Baish, Lance L Munn

**Affiliations:** Department of Material Science and Technology, Guilin University of Electronic Technology, Guilin 541004, China; Department of Material Science and Technology, Guilin University of Electronic Technology, Guilin 541004, China; Department of Radiation Oncology, Massachusetts General Hospital and Harvard Medical School, Boston, MA 02129, USA; Biomedical Engineering, Bucknell University, Lewisburg, PA 17837, USA; Department of Radiation Oncology, Massachusetts General Hospital and Harvard Medical School, Boston, MA 02129, USA

**Keywords:** lymphatic vessel pumping, edema, cell transportation, cell compliance

## Abstract

The lymphatic system plays a vital role in maintaining fluid balance in living tissue and serves as a pathway for the transport of antigen, immune cells, and metastatic cancer cells. In this study, we investigate how the movement of cells through a contracting lymphatic vessel differs from steady flow, using a lattice Boltzmann-based computational model. Our model consists of cells carried by flow in a 2D vessel with regularly spaced, bi-leaflet valves that ensure net downstream flow as the vessel walls contract autonomously in response to calcium and nitric oxide levels regulated by stretch and shear stress levels. The orientation of the vessel with respect to gravity, which may oppose or assist fluid flow, significantly modulates cellular motion due to its effect on the contraction dynamics of the vessel, even when the cells themselves are neutrally buoyant. Additionally, our model shows that cells are carried along with the flow, but when the vessel is actively contracting, they move faster than the average fluid velocity. We also find that the fluid forces cause significant deformation of the compliant cells, especially in the vicinity of the valves. Our study highlights the importance of considering the complex, transient flows near the valves in understanding cellular motion in lymphatic vessels.

**Significance Statement**

The transit of cells through the lymphatic system is an important process in immune responses. Advection of cells in the lymph fluid is conceptually different than that in the blood circulation because of the lack of erythrocytes, the contraction-driven flow, and the intraluminal valves. In this study, we develop a mathematical model to better understand the dynamics of cell transport through the lymphatic system. The simulations show that when vessel contractions drive flow, the average cell velocity can exceed the velocity of the surrounding fluid due to complex fluid dynamics near the valves. By simulating deformable leukocytes, we show that the cell undergoes significant deformation during vessel contractions and when transiting the valves—a result relevant to immune cell mechanobiology.

## Introduction

The lymphatic system is responsible for fluid homeostasis and transport of cells critical to the immune system. It is also a route for cancer cell metastasis. When cells transit blood microvessels, they are exposed to relatively constant shear stress levels. In the lymphatic system, vessel contractions create large temporal and spatial variations in intralumenal shear stress, with peak levels ∼20× the average shear stress (1). In addition, there is significant retrograde flow due to downstream contractions, as upstream valves are closing. Cells traversing the lymphatic system, therefore, experience large inhomogeneous shear stress forces that can mechanically deform the cells and nucleus and influence transit times (2).

Lymph transport and fluid homeostasis are affected by gravitational forces and limb orientation, and a common therapy for peripheral edema is limb elevation so that gravity can assist the drainage. However, there are still outstanding questions concerning how lymphatic function and cell transport are affected by different body forces (3–8). In previous work, we have analyzed leukocyte dynamics in blood flow (9). We have also investigated how the dynamics of nitric oxide (NO; produced by shear stress on lymphatic endothelial cells) and intracellular calcium fluxes in lymphatic muscle cells can establish feedback that controls lymphatic contractions in response to gravitational forces (i.e. limb position) (10–14). In this study, we use a lattice Boltzmann computational model to investigate how lymphatic vessel contractions drive cell transport.

### Model summary

**Fig. 1.**
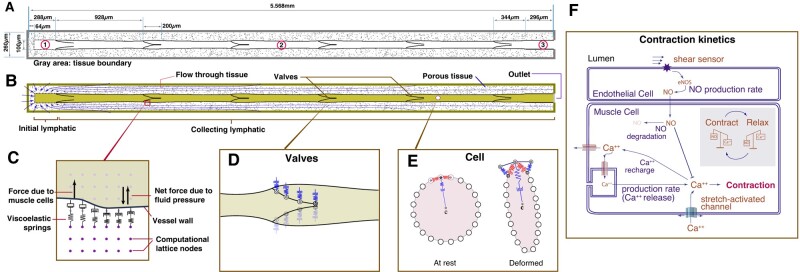
A schematic of a lymphatic vessel and tissue domain for the model (14). A) The simulation domain includes multiple lymphatic components. (1) The initial lymphatic vessel segment, which is connected to the collecting lymphatic lymphangion at right. (2) The collecting lymphatic vessel segment, containing five lymphangions. (3) The outlet section. The surrounding tissue is porous with solid tissue islands with a partial bounce-back boundary condition (15). To anchor and stabilize the outlet region of the collecting vessel, we impose a 0.296-mm length of a noncontracting vessel extending to the outlet boundary. The total vessel length is 5.6 mm. We assume that the surrounding tissue does not deform: when the vessel wall expands and moves into the tissue space, we mathematically replace the tissue with fluid at those nodes (16). The newly formed fluid nodes are assigned based on extrapolation from neighboring nodes in the LBM lattice. As the wall recedes, this process is reversed. B) A schematic of the domain, extravascular flow streams, and a depicted cell. C–E) Schemas for a vessel wall, valve mechanics, and cell mechanics, respectively. F) Simplified Ca^++^ and NO dynamics assumed in the model.

Details of the model have been described elsewhere (11, 14) and are included in the Supplementary material. In summary, the model domain includes a lymphatic vessel system, which consists of an initial lymphatic vessel segment that receives fluid from the surrounding tissue (Fig. 1A, circled “1”). This is connected to several collecting lymphangions arranged in series (Fig. 1A, circled “2”) and one outlet lymphangion (Fig. 1A, circled “3”). Each lymphangion is bounded by two valves, and the vessel is situated in a porous tissue space where pressure gradients can cause fluid movement. The outer boundary of the model domain, represented by a gray line, marks the interface with the surrounding tissue and is modeled as an equilibrium distribution function.

Fluid dynamics are simulated by the D2Q9 lattice Boltzmann method (LBM) (17), and the fluid exchanges momentum with solid elements of the domain, including the vessel wall, valves, cells, and the porous media of the tissue. The fluid moves into the domain from the surrounding tissue across the boundary, as shown in Fig. 1B; the tissue is modeled as a porous medium. Fluid can then enter the initial lymphatic capillary through apertures, which represent primary valves.

The initial lymphatic capillary in our model contains permeable gaps that allow fluid to enter the vessel. These gaps, which make up 50% of the vessel wall, permit fluid to flow freely into the vessel. However, if the pressure gradient favors backflow into the tissue, 15% of the fluid passes back to the tissue, while 85% is reflected. This retrograde flow is necessary to allow closure of the first intraluminal valve during a contraction. At the vessel outlet, we impose a pressure boundary condition (18).

The initial lymphatic segment connects to the collecting lymphatic vessel—consisting of a series of lymphangions separated by explicit mechanical valves. The vessel wall in this region is flexible and is anchored to the surrounding tissue with viscoelastic springs oriented perpendicular to the vessel wall (11). Fluid pressure gradients across the wall induce movement of the vessel wall locally, implemented by transferring momentum between the fluid and wall nodes (Fig. 1C) (11, 16). The bi-leaflet valves are modeled as viscoelastic solid structures anchored at the vessel wall (Fig. 1D) (11). The opening and closing of the valves are passive, entirely controlled by forces imparted by the flowing fluid. In the absence of fluid flow, the valves are biased in the open position, consistent with experimental observations (19).

The cell is modeled as a flexible membrane that encloses fluid with the same density as the surrounding fluid. The cell membrane has bending and stretching elasticity, including elasticity anchored to the center of the cell. The cell membrane is divided into 20 segments, and elastic energy variation is used to calculate the elastic forces stored in the cell membrane (Fig. 1E). By calculating the elastic energy stored in each membrane “spring” and summing this over the entire membrane, we get an overall cell elastic energy, which provides a measure of the deviation of the cell shape from baseline (circular). This metric does not presume any specific deformation geometry. The cell is initially placed 0.0008 cm above the centerline of flow at the entrance to the vessel.

Lymphatic muscle contractions are implemented using a simplified scheme for calcium dynamics in the muscle cells (Fig. 1F) (10). Ca^++^ is restricted to the vessel wall but can diffuse laterally (simulating gap junction transport). It is produced at a baseline level and degrades with first-order kinetics. Additional terms account for calcium-induced Ca^++^ channels and for the rapid accumulation of Ca^++^ in response to increased vessel diameter (to simulate stretch-activated ion channels; see Supplementary material for model details). Contractions are initiated when the Ca^++^ level exceeds a threshold, and the resulting contraction force is proportional to the Ca^++^ level. Ca^++^ is depleted from the cytoplasm according to a recharge rate. As the wall moves, it transfers energy to move the fluid.

The Ca^++^ dynamics described above are known to be modulated by NO production (10, 20), which is affected by fluid shear stress exerted on the endothelial structures (vessel wall and valves). In the model, the NO production rate is proportional to the shear stress at the vessel wall (10, 20), and it degrades exponentially with a half-life of 0.31 s. NO diffuses and convects freely in the domain, and increases the degradation rate of Ca^++^.

To examine the range of behaviors of lymphatic transport that would be expected in vivo, we also consider the effects of gravity, which have a significant influence on contraction dynamics. To simulate gravity, a body force is applied to the fluid, either assisting or opposing the flow. Note that no model parameters are changed when simulating the different gravitational forces (Table S1). The observed changes in contractions naturally emerge due to the mechanobiological mechanisms that drive NO and Ca^++^ dynamics. To initiate the simulations, we start with a high Ca^++^ concentration (just below the threshold level for contraction) at every vessel node. This is sufficient to induce rhythmic contractions, except in cases with high adverse gravity. In these cases, an additional perturbation in the form of a transiently increased Ca^++^ force constant is needed (Fig. S1). Additional details of the mathematical model are included in the Supplemental Material.

## Results

### Effects of gravity and NO production on cell transport

**Fig. 2.**
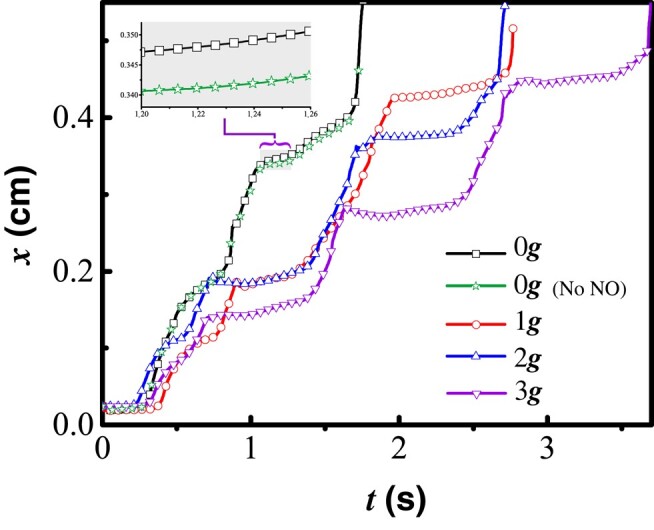
Cell trajectories over time with 0*g*, 1*g*, 2*g*, and 3*g* opposing gravity. The inset shows the detail for *t* ≈ 1.66 s, highlighting the small difference between cells with and without NO generation.

The model cell is introduced within the initial lymphangion at *t* = 0. Because adverse gravitational forces influence lymphatic contractions, we simulate cases with various levels of opposing gravity. Figure 2 shows the position of the cell over time. When moving against 3*g*, the cell periodically reverses direction as it advects because the flow is occasionally retrograde. This occurs when the cell has passed a valve, and a vessel wall contraction occurs in the same lymphangion. When this happens, the cell can also collide with the upstream valve as it closes, causing the cell to pause, thus interrupting its retrograde motion. Adverse gravitational forces also result in elevated transwall pressure, which limits the contraction amplitude. Consequently, cell transit is slower with higher gravity because of the opposing pressure gradient and the backflow that occurs before the valves close.

There are reports that leukocytes can produce NO as they travel in the bloodstream (21, 22). To test whether this would affect lymphatic contraction dynamics and cell velocities in lymphatic vessels, we simulated a cell producing NO. We assume that NO production is sensitive to the average fluid shear stress at the cell surface. We see that the cell moves slightly faster if it is able to produce NO (see the inset panel, Fig. 2). This is likely due to a delay in contraction caused by the additional cell-produced NO. However, the difference in average speed is small and likely of little practical consequence in relatively large diameter vessels.

**Fig. 3.**
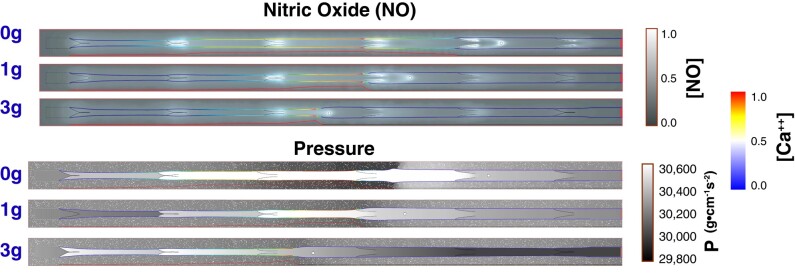
Simulation snapshots of NO, pressure distribution, and Ca^++^ concentrations. In each simulation, there are multiple contraction sequences, and for the snapshots shown, the second vessel contraction is approaching the cell. The cell is shown in white. Top: normalized NO concentrations are shown in the grayscale color map. Bottom: pressure is shown in the grayscale colormap. In both panels, the vessel wall colormap corresponds to the normalized Ca^++^ concentration. The propagation of the Ca^++^ wave is plotted below the vessel in red. Shown are snapshots from simulations with 0*g*, 1*g*, and 3*g* opposing flow. For full simulations, see Videos S1–S6.

To further investigate the factors that affect cell motion, we next analyzed the NO and pressure distributions as a contraction approaches the cell (Fig. 3). The concentration of NO decreases with increased gravity because of the larger vessel diameter and slower flow caused by the gravity-induced pressure; this decreases wall shear stress as well as the resulting NO production. Consistent with experiments (23), the concentration of NO is always higher near the valves and varies during the contraction cycle. As the vessel is contracting, the NO concentration increases near any flanking valve that is open (upstream or downstream). The valve opening sequence along the chain generally follows the direction of flow—from upstream to downstream. When the vessel is relaxing and the fluid is being pulled in (Fig. 3, top), the NO concentration near the upstream valve is higher than the downstream valve. Note that fluid pressure in the tissue outside the vessel decreases transiently as the adjacent vessel contracts (Fig. 3, bottom).

### Cell deformation in flow

**Fig. 4.**
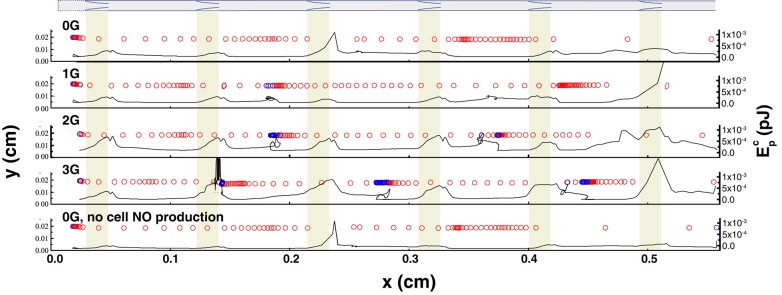
Cell movement and shape in contracting lymphatic vessels. The diagram at the top and the shaded rectangles show the locations of the valves. The black solid lines are the cell’s potential strain energy $E_{p}^{c}$. The outlined circles track the cell trajectory, plotted at 1.33 × 10^−2^ second intervals. The lighter circles (red) are in forward motion, and darker circles (blue) are in retrograde motion. Four cases are shown: without gravity (0*g*), 1*g*, 2*g*, and 3*g*, and without gravity and without NO generation by the cell (see Videos S1–S6).

We next investigated the effects of cell compliance by allowing a nominally circular cell to deform dynamically in response to the local flow field (9). Cell shapes and trajectories are shown as time-resolved snapshots along the length of the vessel in Fig. 4. The larger spaces between snapshots indicate higher velocities, and cells that are moving backward are shown in blue. Fluid forces imparted to the cell cause it to deform, and we can calculate the energy stored in these interactions by determining how the cell changes shape. Specifically, we calculate the deviation of each elastic component from its rest position; this yields a transient potential strain energy, $E_{p}^{c}$, which results from the deformation (see Supplemental Material for details of implementation). When the vessel is relaxing, $E_{p}^{c}$ is approximately 0, indicating that the cell is close to its rest configuration (black line, Fig. 4). When the cell is passing a valve, $E_{p}^{c}$ can spike, indicating significant cell deformation (see Fig. 4, “0G” and “0G, no cell NO production” downstream of the third valve and Fig. 4, “1G” downstream of the sixth valve). This is due to acceleration of the fluid in this region and the increased shear stresses.

Deformations also arise during retrograde motion. When a contraction occurs downstream of the cell, the upstream valve closes, and the cell is flattened because the contraction creates a backward jet of flow between the closing valve leaflets that briefly decelerates the cell (Fig. 4; see Videos S3–S6). These transient deformations as the cell transits the lymphatic system may have implications for the activation of mechanosensitive pathways in the cell. Simulations of cells with different membrane rigidity show that cells with higher deformability undergo more pronounced shape changes, as expected (Videos S7–S9).

**Fig. 5.**
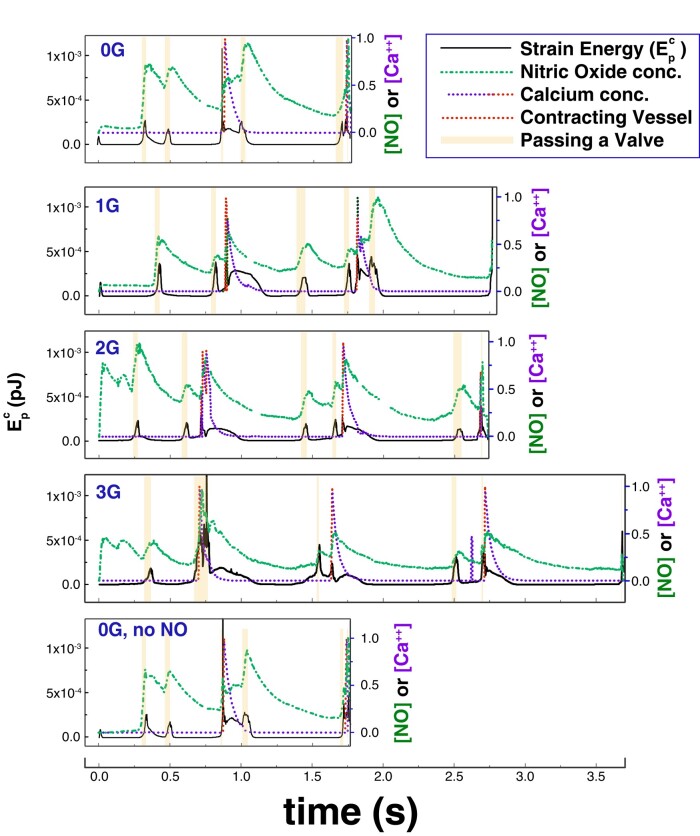
Calcium concentration, NO concentration, and cell potential energy $E_{p}^{c}$ for an advecting cell. $E_{p}^{c}$ is shown by the solid line. The green dash-dot line and purple dashed line are NO and calcium concentrations at the cell location. Four cases are shown: without gravity (0*g*), 1*g*, 2*g*, and 3*g*, and without gravity and without NO generation by the cell (0*g*, no NO). The red portions of the Ca^++^ plot indicate that the vessel is starting to contract. The shaded boxes mean that the cell is passing a valve.

We can also examine how $E_{p}^{c}$ is affected by vessel contractions by plotting local NO and Ca^++^ concentrations in the reference frame of the cell as it transits the vessel (Fig. 5). We again see cell deformation as the cell passes through valves (Fig. 5, shaded boxes). Spikes in $E_{p}^{c}$ also occur when the calcium concentration reaches its peak value near the cell (Fig. 5, red trace), indicating that the initiation of a wall contraction at the cell location is responsible for the deformation. The largest deformations occur when a contraction occurs near the cell as it passes a valve. The short duration of these large deformations indicates that the cell experiences large fluid forces only briefly, and the cell is otherwise exposed to low shear stress. Comparing Fig. 5 cases for 0*g* and 0*g*, no NO for *t* ≈ 0.86 s and *t* > 1.6 s shows that cells generating NO are protected somewhat from large deformations as the local vessel walls contract less forcefully. This also promotes faster cell movement through the valves (see Videos S1–S6).

### Cell and fluid velocities

**Fig. 6.**
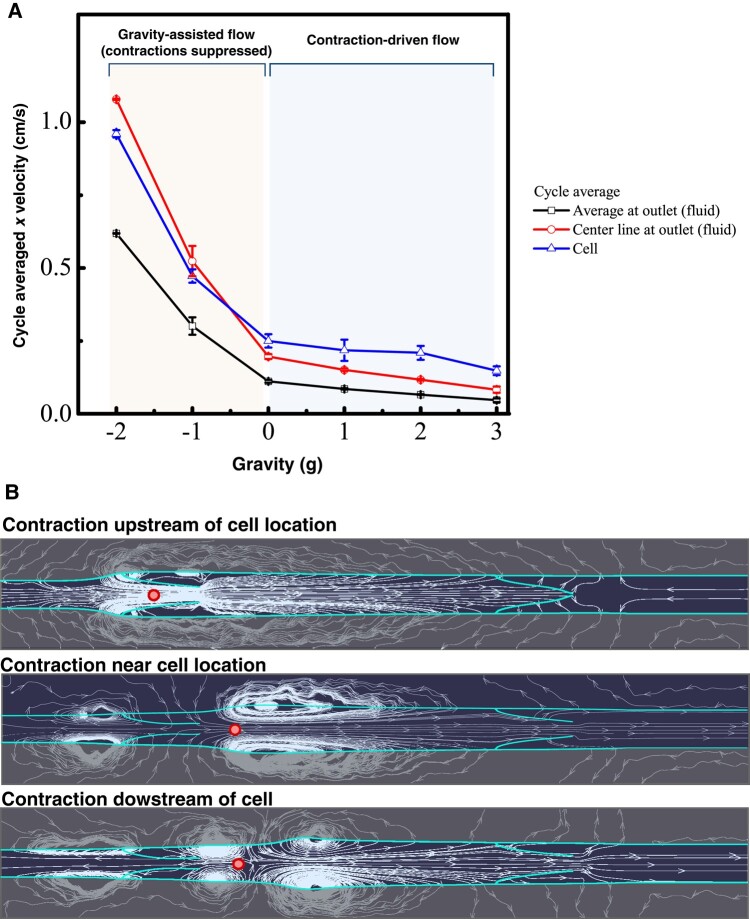
Average velocities and streamlines during cell transit. A) Average cell velocity and average bulk and centerline fluid velocities at the outlet as a function of gravitational force. B) Fluid streamlines while the vessel is contracting (simulation with 3*g* opposing gravitational force). The vessel boundary and valves are highlighted, and the cell location is the circle within the vessel. Snapshots are shown when the contraction occurs before the cell, close to the location of the cell, and downstream of the cell.

We next compared the cell velocity with that of the fluid (Fig. 6A). Because of the periodic contractions and the intraluminal valves, fluid dynamics and velocities are constantly changing, and therefore, we measured the average cell velocity as the distance traveled divided by the total transit time. For the fluid, we calculated the average centerline and bulk fluid velocities at the exit. When negative gravity assists flow (left side regime, Fig. 6A), the cell moves at roughly the same speed as the centerline of the flow. Note that for steady laminar flow between stationary parallel walls, the ratio of centerline velocity to average velocity is 3:2. In our system, the cell velocity approximates this 3:2 ratio when gravity assists the flow, likely due to the suppression of contractions by increased NO production. Indeed, the vessel never contracts when gravity is −2*g* and only occasionally contracts at −1*g*. The intermittent nature of the contractions at −1*g* also contributes to the larger variation in speeds.

On the other hand, when gravity opposes flow (positive gravity), the vessel spontaneously contracts to drive flow. In this regime, the average cell velocity is up to three times faster than the calculated average fluid velocity at the exit—and even faster than the centerline velocity. This result is due to the decreased average diameter of the vessel due to the contractions (which increases cell and fluid centerline velocities during transit) and the fact that the vessel diameter at the exit is fixed at the larger, baseline diameter. Thus, the “effective” vessel diameter for cell transit is affected by the vessel contractions as well as the constrictions formed by the valves. This is most easily visualized by the flow streamlines, which show the converging regions of acceleration as well as the zones of recirculation near the valves (Fig. 6B). Nevertheless, this analysis shows that the cell transits the vessel efficiently during—and in the absence of—contractions.

## Discussion

The lymphatic system is a complex, self-regulated system that drains fluid, cells, and proteins from tissues. Cells move through the lymphatic system as they transit from tissues to lymph nodes and back to the systemic blood circulation. This process is critically important for immune function and also has implications for metastatic dissemination of cancer cells. However, transport in lymphatics—where cell density is sparse, and periodic vessel contractions drive the flow—differs from that in the blood system, where cell densities are high, and there is negligible retrograde flow. Here, we used a mathematical model that includes computational fluid dynamics, accurate mechanobiological vessel dynamics, and fluid–cell interactions to investigate lymphatic transport of cells.

Consistent with previous work (14), our simulations show that gravitational forces affect the dynamics of lymphatic contractions. When gravity assists flow, vessel contractions are less frequent or absent, as gravity-driven flow creates vessel wall shear stress that increases NO production; this, in turn, relaxes the lymphatic muscle cells, inhibiting contractions. In contrast, when flow opposes gravity, contractions drive flow through valves, which maintain an averaged positive flow. During these contractions, there is some retrograde flow that influences cell transport (24). Cell transport is affected by these dynamics, and we see that, in general, cell velocities are higher with assisting or 0*g* and lower when flow is against the direction of gravity. These results suggest that in addition to alleviating edema, therapeutic limb elevation can enhance the transport of immune cells.

We also tested the hypothesis that cell-produced NO might influence the vessel dynamics, and consequently, cell transport speeds. In these simulations, the cells produce NO proportional to shear stress at the cell surface (25), and this NO can diffuse and convect in the fluid phase to reach the vessel wall. The results predict that cell-produced NO has a very small effect on vessel contractions, which causes a correspondingly small increase in cell velocity. Further investigations are needed to see whether this small effect is of any cumulative consequence as the cell moves through the entire lymphatic system.

A major difference in fluid dynamics between the blood and lymphatic systems is the frequent oscillations that occur during lymphatic pumping. Although large arteries in the blood system can have pulsatile flow, oscillations and retrograde flow are generally not observed. In the lymphatic system, the oscillations can have a large effect on the forces felt by transiting cells. Our simulations show that as flow reverses in the vicinity of a cell, there is an observable deformation of the cell membrane. Cell deformation is also exacerbated during transit through valves, where there is flow acceleration due to the partial constriction imposed by the valve leaflets. These cell deformations, which are quickly induced, but decay with a half-life of ∼0.2 s (Fig. 5), may have important implications for cell mechanobiology, as many stress-related pathways can be induced by mechanical forces.

When comparing cell velocities with those of the fluid, we confirmed that cell velocity is roughly the same as fluid velocity in the absence of vessel pumping. When the vessel is contracting, the cells still move faster than the fluid, on average. This result can be explained by the decreased effective diameter of the vessel through which the cell moves, caused by the contractions and the valves. Thus, the ratio of cell to fluid velocity is higher than expected for a noncontracting vessel with a constant baseline diameter.

## Conclusion

Our simulations show that gravitational forces and contraction-induced retrograde flow can affect cell transport velocities and deformation. These effects can, in turn, influence a number of physiological processes, including immune system efficacy and cancer cell dissemination. Future experimental studies may provide more information about cell mechanobiology in lymphatic flow and the transport of immune cell populations at a larger scale through the lymphatic system.

## Supplementary Material

pgae195_Supplementary_Data

## Data Availability

The code used in this study will be made available at https://github.com.
