# Supplementary material for: Fluid dynamics and leukocyte transit in the lymphatic system
Source: PNAS Nexus. 2024 May 17;3(6):pgae195. doi: 10.1093/pnasnexus/pgae195 (PMC11141778; doi:10.1093/pnasnexus/pgae195)
Supplement: pgae195_Supplementary_Data [file pgae195_supplementary_data.zip › Supplement Text Final.pdf]

Supplementary Information for

Fluid dynamics and leukocyte transit in the lymphatic system

Huabing Li, Jingjing zhang, Timothy P. Padera, James W. Baish and Lance Munn.

Lance Munn:
E-mail: munn@steele.mgh.harvard.edu

This PDF file includes:

Figs. S1 to S2
Table S1
Legends for Movies S1 to S9
SI References

Other supplementary materials for this manuscript include the following:

Movies S1 to S9

Methods

Vessel membrane and the valve. Both the membrane and the valve are discretized into segments, which can only move along the y (vertical) direction. They both have bending force:

$$F_B = -K_B((y - y_m) + (y - y_n)), \quad [1]$$

where y_m and y_n are the y-positions of the neighboring segments. For the vessel K_B is constant, but for the valve, it's strong near the anchor point and softer at the tip (1), thus we assume the bending rigidity for the valves as:

$$K_B^v = \frac{2(K_0^v - K_R^v)}{1 + \exp(Ai/n)} + K_R^v, n \geq i \geq 0, \quad [2]$$

where i indicates segment number and n is the total number of valve segments. K_0^v is the maximum of K_B^v at anchor point. K_R^v is an approximation of the minimum of K_B^v, the coefficient A adjusts the soft range of the valve. Elastic force from the tissue:

$$F_E = -K_E(R - R_0), \quad [3]$$

where R is the radius of the vessel or valve, R_0 is the their rest radii. For the vessel, R_0 is a constant, but for the valve, to reproduce the rest curved shape seen in experiments, we assume the valve has a rest shape given by:

$$y_0 = y_{l0} \pm \sqrt{(x - x_0)/B}, \quad [4]$$

where y_{l0} is the rest position of the vessel, x_0 is the anchor point of the valve. '-' and '+' indicate upper and lower valve leaflets. B can adjust the rest position to be biased to stay open or close. The vessel has a minimum radius R_l to avoid excessive contractions. The valve also has a limit position to avoid opening too much, imposed by B. Considering both the vessel and valve leaflets as viscoelastic, a viscous resistance force is introduced as:

$$F_r = -K_r v. \quad [5]$$

The minus sign means that F_r always acts in the direction opposing wall velocity v.

There are lymphatic muscle cells (LMCs) on the lymphatic vessel, which can contract when calcium spreads into the cytoplasm of LMCs. The lymphatic muscle force depends on the concentrations of Ca^{++} and nitric oxide according to (2)):

$$F_M = K_M(\frac{C_{Ca}}{1 + C_{Ca}})(\frac{2R}{R + R_{Ca}})(\frac{1}{1 + K_{NO}C_{NO}}), \quad [6]$$

where C_{Ca} and C_{NO} are the concentrations of calcium and NO respectively, and K_M is the coefficient determining the strength of action. Ca^{++} production and diffusion can be described as (2–7):

$$\begin{aligned} \triangle C_{Ca}(\boldsymbol{x}, t) = \quad & D_{Ca}\nabla^2 C_{Ca}(\boldsymbol{x}, t)\triangle t \\ & +(-K_{Ca}^-(1 + K_{Ca,NO}C_{NO})C_{Ca} + K_{Ca}^+ \\ & +K_{Ca}^+(\frac{(R - R_l)}{(R_{Ca} - R_l)})^{11} \\ & +10K_\delta^+ \delta \uparrow (C_{th}, C_{Ca}))\lambda \triangle t. \end{aligned} \quad [7]$$

Production and diffusion of nitric oxide are simulated through:

$$\begin{aligned} \triangle C_{NO}(\boldsymbol{x}, t) \quad &= D_{NO}\nabla^2 C_{NO}(\boldsymbol{x}, t)\triangle t - \boldsymbol{u} \cdot \nabla C_{NO}(\boldsymbol{x}, t)\triangle t \\ & +(-K_{NO}^- C_{NO}(\boldsymbol{x}, t) + K_{NO}^+ \mid \frac{\partial v_l}{\partial x_n} \mid)\lambda \triangle t, \end{aligned} \quad [8]$$

where C_{NO} is the concentration of NO. D_{NO} is the diffusion coefficient of NO. Each segment moves according to the Newtonian law calculated by a so-called half-step 'leap-frog' scheme (8).

Cell. For convenience, we use energy variation to calculate the internal elastic force of a cell. The cell membrane is divided into twenty segments, each of which has two elastic forces: bending and stretching elastic forces. The potential energy of the membrane can be calculated as:

$$\begin{aligned} E_p^c = \quad & \sum_i (\frac{1}{2}K_S^c((\boldsymbol{p_i} - \boldsymbol{p_{i-1}})^2 + (\boldsymbol{p_i} - \boldsymbol{p_{i+1}})^2) \\ & +\frac{1}{2}K_B^c(H - 1/r)^2, \end{aligned} \quad [9]$$

where, K_S^c and K_B^c are stretching and bending elastic moduli, respectively. H is the curvature of the membrane at point $\boldsymbol{p_i}$ and r is the rest radius of the cell. The elastic force can be calculated by

$$f_x = -\frac{\delta E_p^c}{\delta x}, f_y = -\frac{\delta E_p^c}{\delta y}. \quad [10]$$

In this calculation, we choose $\delta x = \delta y = 10^{-10}$ lattice unit. The membrane of a cell is also viscoelastic. Considering the cytoskeletal proteins, the viscous force of a segment can be calculated as:

$$F_r^c = -K_r^c v, \qquad [11]$$

where v is the velocity relative to the cell membrane mass center. The hydrodynamic force on point $\boldsymbol{p_i}$ is the average hydrodynamic force acting on two neighbor segments. $\boldsymbol{p_i}$ moves according to the Newtonian law. K_r^c is related to the mechanics of or the cytoskeleton and nucleus. In order to keep the cell area approximately constant, we use a feed back process of density as: $\Delta\rho = -(s - s_0) * \rho_0/5.0/N$. s and s_0 are the current and initial cell area, ρ_0 is the initial fluid density inside the cell, and N is the number of fluid nodes inside the cell. $\Delta\rho/9$ is added to each fluid node inside the cell as well as to the 9 distribution functions. The boundary conditions on the cell membrane, valve leaflets, and the vessel wall use a so called curved boundary condition (9), and the hydrodynamic forces on those boundaries are calculated using the so-called stress integration method (10)

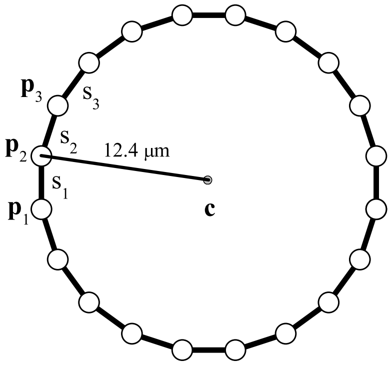

Fig. S1. A cell with rest radius of $r = 12.4\mu m$ is divided into twenty segments. s_i indicates i segment and $\boldsymbol{p_i}$ is its connection to the previous segment. **c** is the center of mass.

Extreme treatment. Extreme treatment is used when one body is too close to a limit position or two bodies are too close to each other. Shown in Fig. S2 (A), if a segment is too close to a limit position, for example, the vessel contracts too much and the gap between the upper membrane and the limit position $(R = R_l)$ $\delta < \Delta$, we multiply F_E by $(\frac{\Delta}{\delta})^{11}$. The valve also has a limit position described by Eq. (4) for when B is maximum. If two membranes are too close to each other (see Fig. S2 (B)), we superpose a repulsive force as $\boldsymbol{F} = k_0((\frac{\Delta}{\delta})^{11} - 1)\boldsymbol{n}$ acting on the segment of one membrane if the gap $\delta < \Delta$. The counterforce acting on the other membrane is divided into $\boldsymbol{F}_1$ and $\boldsymbol{F}_2$; where $\boldsymbol{F}_1 = -\frac{d1}{d1+d2}\boldsymbol{F}$, $\boldsymbol{F}_2 = -\frac{d2}{d1+d2}\boldsymbol{F}$, here we choose $k_0 = 0.00001$ lattice unit. As in Fig. S2 (C), if the vertical intersection is not inside the segment, a repulsive force should act on the adjacent points. If a valve is closing and the two leaflets of the valve are too close, extreme treatment also includes a lubrication force (11) to stabilize the membranes of the valve when there is no fluid node between them. In our simulation, the Newtonian time of the vessel and valve is 1/100 of lattice Boltzmann time step. For the cell, we use dynamic accuracy, and specify that the Newtonian time step of the cell depends on the moving speed of the cell. For example, if the cell speed is smaller than 3 cm/s, the cell's Newtonian time step is the same as valve and vessel wall; otherwise, if the cell speed is larger than 3 cm/s and smaller than 3.6 cm/s, the cell's Newtonian time step is 1/5 of that of the valve and vessel wall. If the speed is larger than 3.6 cm/s, the time step of the cell is decreased further. Because lymphatic flow is pulsatile, the cell can accelerate to high speeds and collide with a valve. When the cell collides with a valve (gap $<$ 1 lattice unit), the cell's Newtonian time step is decreased to 1/45 of that of the valve and vessel wall. This schema increases computational efficiency while optimizing accuracy of the simulations.

Simulation parameters

The diameter of the vessel at rest is assumed to be $D' = 0.01cm$. The lymph inside is treated as water with kinematic viscosity of $\nu' = 0.01cm^2/s$ and density of $\rho' = 1g/cm^3$. The densities of valves and the cell are also treated as water, but the density of the vessel is eighty times that of water. In simulations, on the lattice, we choose a vessel diameter of $D = 25$, and a relaxation time $\tau = 0.75$; thus the kinematic viscosity $\nu = (2\tau - 1)/6 = 0.0833$, density at inlet is $\rho = 1$, initial fluid density $\rho_0 = 1$, and initial velocity is zero. The initial calcium concentration at the wall is 0.0999, which is close but below the threshold for calcium fluxes. Initial NO concentration on each lattice is zero. So each time step is $T = \frac{\nu}{\nu'}(\frac{D'}{D})^2 = 1.33 \times 10^{-6}s$, each lattice length is $L = \frac{D'}{D} = 0.0004cm$, and the pressure unit is $P = (L/T)^2 \text{g} \cdot \text{cm}^{-3} = 9.045 \times 10^4 \text{g} \cdot \text{cm}^{-1} \cdot \text{s}^{-2}$. Other parameters are given in table S1. Here, we take advantage of parallel computing by solving multiple lymphangions using MPI. When discretizing the membranes of the vessel and valves, we set the length of each segment to two lattice units, but 0.9738 lattice unit for the cell.

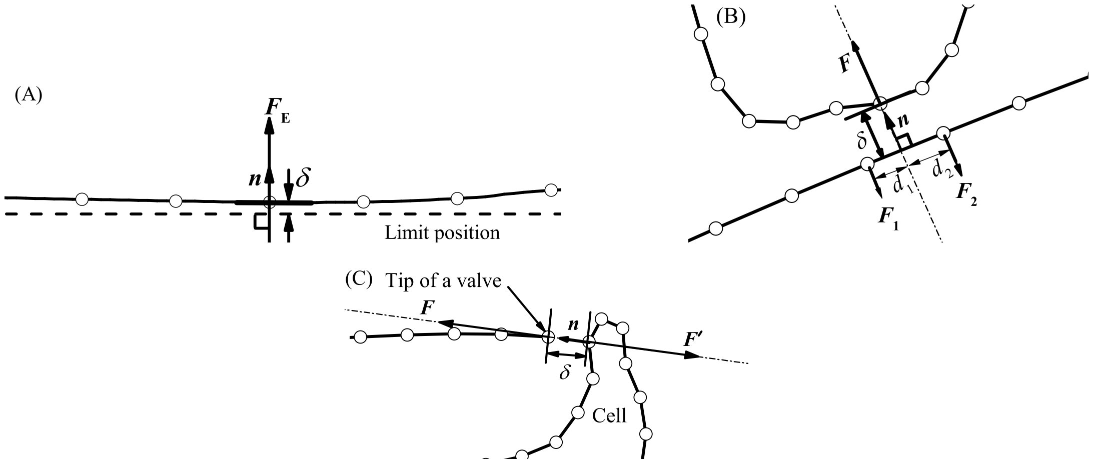

Fig. S2. Schematic diagram of (A) a membrane goes too close to a limit position, (B) Two membrane come too close, (C) The tip of a valve leaflet goes too close to a cell. δ is the gap between two membranes or a membrane and the limit position. $\boldsymbol{F}$ is a rapidly increasing force as δ decreases. $\boldsymbol{F_1}$, $\boldsymbol{F_2}$ and $\boldsymbol{F'}$ are the counterforce of $\boldsymbol{F}$. $\boldsymbol{n}$ is the unit vector.

Parameter	Definition	Value	Units	Source
**Chemical properties of NO and** Ca^{++}				
D_{NO}	NO diffusivity	1.2×10^{-4}	cm^2/s	$3.0 \times 10^{-5}(\mathrm{cm}^2/\mathrm{s})$(12)
K^-_{NO}	NO degradation rate constant	75.1	s^{-1}	Estimated
K^+_{NO}	NO production rate constant	20	Dimensionless	Estimated
D_{Ca}	Ca^{++} diffusivity	5.02×10^{-7}	cm^2/s	$6.5 \times 10^{-7} cm^2/s$ (13)
K^-_{Ca}	Ca^{++} degradation rate constant	375.9	s^{-1}	Estimated
K^+_{Ca}	Ca^{++} production rate constant	22.6	s^{-1}	Estimated
K^+_{δ}	Ca^{++} production rate constant	1.1×10^5	s^{-1}	Estimated
C_{th}	Ca^{++} threshold	0.1	Dimensionless	Estimated
R_{Ca}	Threshold radius for Ca^{++} channel sensitization	$R_0 - 0.5$	L	Estimated
$K_{Ca,NO}$	Rate constant for NO inhibition of Ca^{++}	0.5	Dimensionless	Estimated
λ	Chemical reaction rate constant	0.03	Dimensionless	Estimated
**VESSEL**				
K_M	Force constant for Ca^{++}	1.0×10^{-4}	$\mathrm{g \cdot cm/s^2}$	Estimated
K_E	Young's elastic modulus of the vessel	407.0	$\mathrm{dynes/cm^2}$	Estimated
K_B	Young's bending modulus of the vessel	3.6×10^4	$\mathrm{dynes/cm^2}$	$10^6(\mathrm{dynes/cm^2})$(14)
K_r	Viscosity coefficient of vessel	4.8×10^{-9}	$\mathrm{g/s}$	Estimated
K_{NO}	NO inhibition of force	0.3	Dimensionless	Estimated
R_l	Limit radius	7.5	L	Estimated
R_0	Rest radius of the vessel	12.5	L	Estimated
**VALVE**				
A	How soft the valve is	6	Dimensionless	Estimated
B	How much the valve biased to open	1500	cm^{-1}	Estimated
K^v_E	Young's elastic modulus of valves	9.0×10^{-4}	$\mathrm{dynes/cm^2}$	Estimated
K^v_0	Young's Bending modulus of the base of valves	7.2×10^4	$\mathrm{dynes/cm^2}$	$10^6(\mathrm{dynes/cm^2})$ (14)
K^v_R	Young's bending modulus of the tip of valves	0.018	$\mathrm{dynes/cm^2}$	$0.1k^v_0$ (1)
K^v_r	Viscosity coefficient of the valve membrane	4.8×10^{-9}	$\mathrm{g/s}$	Estimated
**VESSEL & VALVE**				
$\triangle$		0.5	L	Estimated
**CELL**				
K^c_S	Stretch modulus of the cell membrane	3.6×10^{-3}	$\mathrm{g/s^2}$	$5 \times 10^{-3} g/s^2$ minimum for red cell (15)
K^c_B	Bending modulus of the cell membrane	3.46×10^{-8}	$\mathrm{g \cdot cm^2/s^2}$	Red cell, 10^{-1} pNμm(16, 17)
K^c_r	Viscosity coefficient of the cell membrane to the mass center	1.92×10^{-6}	$\mathrm{g/s}$	Estimated

Table S1. Chemical parameters of NO and Ca^{++}; Mechanical parameters of the fluid, vessel wall, valves and the cell.

Movie S1. Lymphatic vessel contraction and cell transportation without gravity (NO).

Movie S2. Lymphatic vessel contraction and cell transportation without gravity (pressure).

Movie S3. Lymphatic vessel contraction and cell transportation with one times gravity opposing (NO).

Movie S4. Lymphatic vessel contraction and cell transportation with one times gravity opposing (pressure).

Movie S5. Lymphatic vessel contraction and cell transportation with three times gravity opposing (NO).

Movie S6. Lymphatic vessel contraction and cell transportation with three times gravity opposing (pressure).

Movie S7. Transport of a cell with baseline membrane rigidity.

Movie S8. Transport of a cell with membrane rigidity reduced to 0.1 times baseline.

Movie S9. Transport of a cell with membrane rigidity reduced to 0.01 times baseline.

References

1. GA Buxton, N Clarke, Computational phlebology: the simulation of a vein valve. *J. biological physics* **32**, 507–521 (2006).
2. C Kunert, JW Baish, S Liao, TP Padera, LL Munn, Mechanobiological oscillators control lymph flow. *Proc. Natl. Acad. Sci.* **112**, 10938–10943 (2015).
3. Y Osipchuk, M Cahalan, Cell-to-cell spread of calcium signals mediated by atp receptors in mast cells. *Nature* **359**, 241 (1992).

4. EA Newman, KR Zahs, Calcium waves in retinal glial cells. *Science* **275**, 844–847 (1997).
5. AC Charles, JE Merrill, ER Dirksen, MJ Sandersont, Intercellular signaling in glial cells: calcium waves and oscillations in response to mechanical stimulation and glutamate. *Neuron* **6**, 983–992 (1991).
6. A Kapela, A Bezerianos, NM Tsoukias, A mathematical model of ca2+ dynamics in rat mesenteric smooth muscle cell: agonist and no stimulation. *J. theoretical biology* **253**, 238–260 (2008).
7. M Jafarnejad, et al., Measurement of shear stress-mediated intracellular calcium dynamics in human dermal lymphatic endothelial cells. *Am. J. Physiol. Circ. Physiol.* **308**, H697–H706 (2015).
8. MP Allen, DJ Tildesley, *Computer simulation of liquids.* (Oxford university press), (2017).
9. R Mei, LS Luo, W Shyy, An accurate curved boundary treatment in the lattice boltzmann method. *J. computational physics* **155**, 307–330 (1999).
10. H Li, X Lu, H Fang, Y Qian, Force evaluations in lattice boltzmann simulations with moving boundaries in two dimensions. *Phys. Rev. E* **70**, 026701 (2004).
11. X Yuan, R Ball, Rheology of hydrodynamically interacting concentrated hard disks. *The J. chemical physics* **101**, 9016–9021 (1994).
12. IG Zacharia, WM Deen, Diffusivity and solubility of nitric oxide in water and saline. *Annals biomedical engineering* **33**, 214–222 (2005).
13. NL Allbritton, T Meyer, L Stryer, Range of messenger action of calcium ion and inositol 1, 4, 5-trisphosphate. *Science* **258**, 1812–1815 (1992).
14. R Wesly, RN Vaishnav, J Fuchs, DJ Patel, J Greenfield Jr, Static linear and nonlinear elastic properties of normal and arterialized venous tissue in dog and man. *Circ. research* **37**, 509–520 (1975).
15. GL HW, M Wortis, R Mukhopadhyay, Stomatocyte–discocyte–echinocyte sequence of the human red blood cell: Evidence for the bilayer–couple hypothesis from membrane mechanics. *Proc. Natl. Acad. Sci.* **99**, 16766–16769 (2002).
16. MM Dupin, I Halliday, CM Care, L Alboul, LL Munn, Modeling the flow of dense suspensions of deformable particles in three dimensions. *Phys. Rev. E* **75**, 066707 (2007).
17. M Dao, CT Lim, S Suresh, Mechanics of the human red blood cell deformed by optical tweezers. *J. Mech. Phys. Solids* **51**, 2259–2280 (2003).
